# Healthcare Worker Acceptance of Pandemic (H1N1) 2009 Vaccination, Morocco

**DOI:** 10.3201/eid1610.100984

**Published:** 2010-10

**Authors:** Rida Tagajdid, Hicham El Annaz, Taoufik Doblali, Kawtar Sefiani, Bouchra Belfquih, Saad Mrani

**Affiliations:** Author affiliations: Mohammed V Military Teaching Hospital, Rabat, Morocco (R. Tagajdid, H. El Annaz, B. Belfquih, S. Mrani);; Mohammed V-Souissi University, Rabat (R. Tagajdid, H. El Annaz, T. Doblali, K. Sefiani, B. Belfquih, S. Mrani)

**Keywords:** Vaccination, pandemic, influenza, healthcare workers, viruses, Morocco, letter

**To the Editor:** In Morocco, the first case of pandemic (H1N1) 2009 was diagnosed on June 12, 2009 (*1*). Because a main determinant of public immunization success is healthcare workers’ support and recommendations and because little is known about such with regard to pandemic (H1N1) 2009 vaccination in Morocco, our aim was to document healthcare workers’ knowledge, attitudes, practices, and acceptance of pandemic (H1N1) 2009 vaccination in Morocco.

From January 15 through February 28, 2010, a structured, self-administered, anonymous questionnaire was distributed to a convenience sample of 1,332 healthcare workers in 5 public hospitals in Rabat, Morocco. Completed questionnaires were analyzed by using SPSS version 10.0 (SPSS, Chicago, IL, USA). The 1,002 responses gave a response rate of 75% (≈17% of the entire staff of the University Hospital of Rabat).

We found that the hospital staff had acquired basic knowledge about transmission and prevention of the pandemic (H1N1) 2009 virus. Responses indicated that 218 (22%) study participants had accepted vaccination (i.e., had been vaccinated) against this virus. Markedly more healthcare workers in Morocco were undervaccinated than were those in the United States; by mid-January 2010, estimated vaccination coverage among healthcare workers was 37.1% (*2*). Some evidence indicates that willingness of healthcare workers to be vaccinated with the new vaccine is poor: 48.0% in Hong Kong Special Administrative Region, People’s Republic of China (*3*) and 22.3% in the United States (*4*). Vaccination coverage was significantly higher for those 20–30 years of age than for those in other age groups (p = 0.001). The analysis by occupational category showed significantly higher coverage for paramedical staff (26%) than for physicians and pharmacists (19%) (p<0.01). The main causes for this reluctance were fear of adverse effects, concerns about the new adjuvant used, the short duration of clinical trials, and influence of the media.

The low acceptance rate of vaccination for pandemic (H1N1) 2009 among healthcare workers in Morocco is alarming because they serve as an example for their patients and the public. Vaccination is needed to keep the healthcare system operating at maximum capacity during a pandemic. The following factors appear to play a major role in acceptance: accessibility of the vaccine within the service; free vaccine; and a display explaining vaccination’s benefits, protective value, and risk for adverse effects (*5**,**6*). Policy makers could use our findings to improve the vaccination strategy for healthcare workers in future vaccination campaigns.
